# Vision Impairment and Subjective Cognitive Decline–Related Functional Limitations — United States, 2015–2017

**DOI:** 10.15585/mmwr.mm6820a2

**Published:** 2019-05-24

**Authors:** Sharon Saydah, Robert B. Gerzoff, Christopher A. Taylor, Joshua R. Ehrlich, Jinan Saaddine

**Affiliations:** ^1^Division of Diabetes Translation, National Center for Chronic Disease Prevention and Health Promotion, CDC; ^2^Division of Population Health, Applied Research and Translation Branch, National Center for Chronic Disease Prevention and Health Promotion, CDC; ^3^Department of Ophthalmology and Visual Sciences, University of Michigan, Ann Arbor, Michigan.

Vision impairment affects approximately 3.22 million persons in the United States and is associated with social isolation, disability, and decreased quality of life (*1*). Cognitive decline is more common in adults with vision impairment (*2*,*3*). Subjective cognitive decline (SCD), which is the self-reported experience of worsening or more frequent confusion or memory loss within the past 12 months, affects 11.2% of adults aged ≥45 years in the United States (*4*). One consequence of SCD is the occurrence of functional limitations, especially those related to usual daily activities; however, it is not known whether persons with vision impairment are more likely to have functional limitations related to SCD (*4*). This report describes the association of vision impairment and SCD-related functional limitations using Behavioral Risk Factor Surveillance System (BRFSS) surveys for the years 2015–2017. Adjusting for age group, sex, race/ethnicity, education level, health insurance, and smoking status, 18% of adults aged ≥45 years who reported vision impairment also reported SCD-related functional limitations, compared with only 4% of those without vision impairment. Preventing, reducing, and correcting vision impairments might lead to a decrease in SCD-related functional limitations among adults in the United States.

This analysis used data from the BRFSS, an annual state-based, cross-sectional telephone survey of noninstitutionalized adults aged ≥18 years, combining data from 2015, 2016, and 2017.* During those 3 years, 208,601 respondents aged ≥45 years in 49 states (all except Pennsylvania), Puerto Rico, and the District of Columbia (DC) completed the optional cognitive decline module.^†^^,^^§^^,^^¶^ For states that administered the module in multiple years, only the most recent year’s data were included. For the BRFSS surveys in 2015, 2016, and 2017, the combined landline and cellular telephone response rates among states, Puerto Rico, and DC ranged from 30.6% to 64.1% (median = 45.7%).

Among all respondents aged ≥45 years, those classified as having SCD responded affirmatively to the question “During the past 12 months, have you experienced confusion or memory loss that is happening more often or is getting worse?” Respondents with SCD were then asked two follow-up questions: 1) “During the past 12 months, as a result of confusion or memory loss, how often have you given up day-to-day household activities or chores you used to do, such as cooking, cleaning, taking medications, driving, or paying bills?” and 2) “During the past 12 months, how often has confusion or memory loss interfered with your ability to work, volunteer, or engage in social activities outside the home?” Responses of “always,” “usually,” and “sometimes” were classified as positive responses, and responses of “rarely” and “never” were classified as negative responses (*4*). Functional limitations caused by SCD were defined as a positive response to either of the two follow-up questions. Vision impairment was defined as a yes response to the question “Are you blind or do you have serious difficulty seeing, even when wearing glasses?” Descriptive analyses examined population characteristics by vision impairment and SCD-related functional limitations status. Covariates included age group (45–64 years, 65–74 years, or ≥75 years); sex (male or female); race/ethnicity (non-Hispanic white, non-Hispanic black, non-Hispanic multiracial, Hispanic, or non-Hispanic other); education level (less than high school, high school graduate or some college, or college graduate); smoking status (never, former, or current); and having health insurance (yes or no). Multivariate logistic regression models were used to calculate predicted marginal proportions and examine the relationship between vision impairment and SCD-related functional limitations, adjusting for age, sex, race/ethnicity, education level, and smoking status. All estimates used the BRFSS-provided sampling weights to account for the complex survey design and nonresponse. Analysis was completed using SUDAAN (version 11.0.3; RTI International).

The overall prevalence of vision impairment among respondents was 6.2% (95% confidence interval [CI] = 6.0%–6.3%), and the overall prevalence of SCD with functional limitations was 5.5% (95% CI = 5.3%–5.7%). The prevalence of vision impairment without functional limitations related to SCD increased with age from 4.4% (95% CI = 4.2%–4.7%) among those aged 45–64 years to 7.2% (95% CI = 6.7%–7.6%) for those aged ≥75 years (Table). Among adults reporting SCD-related functional limitations without vision impairment, the proportion in each of the three age groups was similar (range = 2.8 [65–74 years] to 4.4 [45–64 years]), as was the age distribution among those with vision impairment (range = 0.9% [age 65–74 years] to 1.8 [45–64 years]). Similarly, no significant differences in report of SCD-related functional limitations among those with and without vision impairment were seen when stratified by race/ethnicity. Vision impairment without SCD related limitations was highest among Hispanics (10.4%, 95% CI = 9.3–11.6) and lowest among non-Hispanic whites (3.6%, 95% CI = 3.5%–3.8%). However, prevalences of vision impairment and SCD-related functional limitations were higher among adults with less than a high school diploma (4.1%, 95% CI = 3.6%–4.6%), who were current smokers (3.6%, 95% = 3.2%–4.0%), and who did not have health insurance (3.0%, 95% CI = 2.5%–3.7%) than among college graduates (0.4%, 95% CI = 0.3%–0.4%), those who had never smoked (0.9%, 95% CI = 0.8%–1.1%), and those who had health insurance (1.4%, 95% CI =1.3%–1.5%).

**TABLE:** Percentage of adults aged ≥45 years who reported vision impairment with and without subjective cognitive decline (SCD)–related functional limitations (FL), by selected characteristics — Behavioral Risk Factor Surveillance System, 49 states, Puerto Rico, and District of Columbia, 2015–2017

Characteristic	Vision and SCD-related FL status, % (95% CI)				
	Overall	No vision impairment, no SCD-related FL	No vision impairment, with SCD-related FL	Vision impairment, no SCD-related FL	Vision impairment, with SCD-related FL
**Overall**	**100**	**89.7 (89.4–89.9)**	**3.9 (3.8–4.1)**	**4.9 (4.7– 5.1)**	**1.5 (1.4–1.6)**
**Age group (yrs)**					
45–64	63.1 (62.8–63.3)	89.4 (89.0–89.8)	4.4 (4.1–4.6)	4.4 (4.2–4.7)	1.8 (1.6–2.0)
65–74	21.6 (21.4–21.8)	91.8 (91.4–92.2)	2.8 (2.5–3.0)	4.6 (4.3–4.9)	0.9 (0.8–1.0)
≥75	15.3 (15.2–15.5)	87.8 (87.2–88.4)	3.9 (3.6–4.3)	7.2 (6.7–7.6)	1.1 (0.9–1.3)
**Sex**					
Male	47.0 (46.8–47.3)	90.1 (89.7–90.5)	3.9 (3.7–4.1)	4.7 (4.4–4.9)	1.3 (1.2–1.5)
Female	53.0 (52.7–53.2)	89.3 (88.9–89.7)	4.0 (3.8–4.2)	5.1 (4.8–5.4)	1.6 (1.5–1.8)
Race/Ethnicity*					
White, non-Hispanic	68.4 (68.1–68.8)	91.7 (91.4–91.9)	3.6 (3.5–3.8)	3.6 (3.5–3.8)	1.0 (0.9–1.2)
Black, non-Hispanic	10.1 (9.9–10.3)	85.4 (84.3–86.3)	4.9 (4.3–5.4)	7.0 (6.2–7.8)	2.8 (2.4–3.3)
Multiracial, non-Hispanic	1.2 (1.2–1.3)	84.6 (82.0–86.9)	5.9 (4.6–7.6)	7.6 (5.9–9.7)	1.9 (1.3–2.8)
Hispanic	13.9 (13.6–14.2)	82.2 (80.8–83.6)	4.5 (3.9–5.2)	10.4 (9.3–11.6)	2.9 (2.3–3.5)
Other, non-Hispanic	6.4 (6.1–6.6)	88.8 (87.1–90.3)	4.4 (3.6–5.4)	5.0 (4.0–6.2)	1.8 (1.2–2.7)
**Education level**					
Less than high school graduate	14.7 (14.4–14.9)	76.5 (75.3–77.7)	7.3 (6.7–8.0)	12.1 (11.1–13.1)	4.1 (3.6–4.6)
High school graduate/Some college	57.0 (56.7–57.3)	89.7 (89.4–90.1)	4.3 (4.0–4.5)	4.5 (4.3–4.8)	1.5 (1.3–1.6)
College graduate	28.3 (28.1–28.6)	95.7 (95.5–95.9)	1.7 (1.6–1.9)	2.2 (2.1–2.4)	0.4 (0.3–0.4)
**Smoking status**					
Never smoked	54.0 (53.7–54.3)	91.8 (91.4–92.1)	3.0 (2.8–3.2)	4.3 (4.0–4.5)	0.9 (0.8–1.1)
Former smoker	31.9 (31.7–32.2)	90.0 (89.5–90.4)	3.7 (3.5–4.0)	4.8 (4.5–5.1)	1.5 (1.3–1.8)
Current smoker	14.1 (13.9–14.3)	81.2 (80.3–82.1)	7.8 (7.2–8.4)	7.4 (6.7–8.1)	3.6 (3.2–4.0)
**Health insurance status**					
Health insurance	93.0 (92.8–93.1)	90.1 (89.9–90.4)	3.8 (3.7–4.0)	4.6 (4.5–4.8)	1.4 (1.3–1.5)
No health insurance	7.0 (6.9–7.2)	83.2 (81.6–84.7)	5.3 (4.5–6.3)	8.5 (7.3–9.8)	3.0 (2.5–3.7)

**Abbreviation:** CI = confidence interval.

* Whites, blacks, multiracial, and other races/ethnicities were non-Hispanic; Hispanic persons could be of any race.

After adjusting for demographics, smoking status, and vision impairment, the prevalence of functional limitations related to SCD was highest among persons aged 45–64 years (6%) and lowest among those aged 65–74 years (4%; p<0.001) (Figure). In addition, non-Hispanic whites and Hispanics reported the lowest prevalences (5%), and non-Hispanic persons of other races reported the highest prevalence (8%) (p<0.001). The prevalence of SCD-related functional limitations among persons having less than a high school education (9%) was three times that of those reporting being a college graduate (3%) (p<0.001). Being a current smoker was associated with a higher prevalence of SCD-related functional limitations (9%), compared with being a former smoker (5%) or a person who had never smoked (4%) (p<0.001). After adjusting for demographics and smoking status, the highest prevalence of SCD-related functional limitations (18%) was among adults with vision impairment; prevalence among those with no reported vision impairment was 4% (p<0.001).

**FIGURE:**
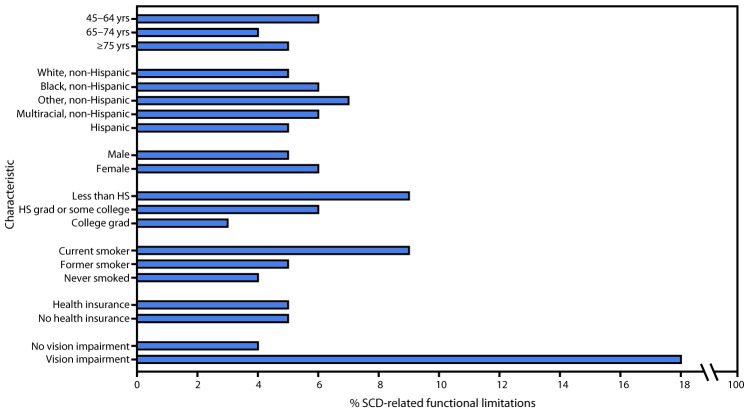
Adjusted percentage* of subjective cognitive decline (SCD)–related functional limitations among adults aged ≥45 years, by demographic characteristics, smoking status, and vision impairment — Behavioral Risk Factor Surveillance System, 49 states,^†^ Puerto Rico, and the District of Columbia, 2015–2017 **Abbreviation:** HS = high school. * Adjusted prevalence based on predicted marginal from logistic regression models adjusting for age, sex, race/ethnicity, smoking status, health insurance status, and vision impairment. ^†^ Excluding Pennsylvania.

## Discussion

Functional limitations have been reported by 50% of adults aged ≥45 years with SCD (*4*), and vision impairment has been reported by 6% (*5*). Previous studies have determined that vision impairment and cognitive decline might co-occur (*2*) and might be causally related (*3*). Recent studies have pointed to changes in the retina as a potential biomarker for dementia (*6*), highlighting the link between vision impairment and cognitive decline. However, the association of vision impairment with SCD-related functional limitations has not been well characterized. This report found that among adults aged ≥45 years, SCD-related functional limitations were three and one half times higher among adults with vision impairment than among those with no vision impairment. The number of adults in the United States with vision impairment is projected to double in the next 30 years (*5*); therefore, understanding the impact of co-occurring vision impairment and SCD on functional abilities is an important public health concern.

Vision impairment might lead to decreased quality of life, functional limitations, and an increased risk of mortality (*7*,*8*). Vision impairment might prevent persons from performing instrumental activities of daily living. However, a previous study found that the relationship between vision impairment and cognitive decline might be modified by a tailored vision rehabilitation program (*9*). Further work can help to determine whether vision rehabilitation is also an effective strategy to improve functional limitations associated with SCD.

Vision impairment can be caused by treatable forms of vision loss such as cataracts and refractive errors, along with age-related macular degeneration, diabetic retinopathy, and glaucoma. Measures to prevent vision impairment and vision loss include receiving eye care and a comprehensive eye exam. Additional ways to protect eyes and prevent vision loss include knowing family history of eye health, eating healthy, maintaining healthy weight, wearing protective eyewear, quitting or never starting smoking, washing hands before removing contact lens, and practicing workplace eye safety.

The findings in this report are subject to at least three limitations. First, these results are based on self-reported vision difficulty and SCD-related functional limitations. Objective measures of cognitive and visual functioning were not administered as part of BRFSS (*4*). Second, response bias might have affected the response to questions on vision impairment, SCD, and functional limitations. For example, older persons might be less likely to report SCD-related functional limitations if they consider them to be part of the aging process, thus reducing the reported prevalence of these limitations in this population. Finally, BRFSS is only administered to noninstitutionalized adults, thereby excluding those living in long-term care facilities where nearly one third of residents might have vision and cognitive impairments (*10*). These limitations might have biased these results toward the null hypothesis and might limit their generalizability across all populations. The strength of this analysis is that it includes nearly all states, Puerto Rico, and DC, representing 253 million U.S. adults.

Vision impairment is an important, growing public health concern in the United States (*5*). Adults with vision impairment might have higher levels of difficulties with activities of daily living (e.g., eating and bathing) and instrumental activities of daily living (e.g., managing finances and using a telephone) (*10*). Having vision impairment might increase the likelihood that persons with SCD report related functional limitations. Addressing vision impairment through prevention or corrective treatment might reduce functional SCD-associated limitations in the adult population.

**Summary**

**What is already known about this topic?**

Vision impairment often co-occurs with cognitive decline, which can be associated with functional limitations. The association between vision impairment and functional limitations related to subjective (self-reported) cognitive decline (SCD) has not been well characterized.

**What is added by this report?**

Analysis of 2015–2017 Behavioral Risk Factor Surveillance System data determined that, after adjusting for age and other demographic and smoking characteristics, 18% of adults who reported vision impairment also reported SCD-related functional limitations, compared with only 4% of those without vision impairment.

**What are the implications for public health practice?**

Prevention or correction of vision impairment might be important in in reducing functional limitations related to cognitive decline in adults aged ≥45 years.

## Footnotes

*
https://www.cdc.gov/brfss .

† https://www.cdc.gov/brfss/annual_data/2015/pdf/2015-sdqr.pdf.

§ https://www.cdc.gov/brfss/annual_data/2016/pdf/2016-sdqr.pdf.

¶ https://www.cdc.gov/brfss/annual_data/annual_2017.html.
